# Associations Among Tremor Amplitude, Activities of Daily Living, and Quality of Life in Patients with Essential Tremor

**DOI:** 10.5334/tohm.877

**Published:** 2024-05-03

**Authors:** Margaret E. Gerbasi, Rodger J. Elble, Eddie Jones, Alexander Gillespie, John Jarvis, Elizabeth Chertavian, Zachary Smith, Mina Nejati, Ludy C. Shih

**Affiliations:** 1 Sage Therapeutics, Inc., Cambridge, MA, USA; 2 Southern Illinois University School of Medicine, Springfield, IL, USA; 3 Adelphi Real World, Adelphi Mill, Bollington, United Kingdom; 4 Medicus Economics, Milton, MA, USA; 5 Biogen, Cambridge, MA, USA; 6 Boston University School of Medicine, Boston, MA, USA; 7 Boston Medical Center, Boston, MA, USA

**Keywords:** essential tremor, upper limb tremor, TETRAS, activities of daily living, quality of life

## Abstract

**Background:**

Essential tremor (ET) is a disabling syndrome consisting of tremor, primarily in the upper limbs. We assessed the correlation of The Essential Tremor Rating Assessment Scale (TETRAS) Performance Item 4 ratings of upper limb tremor with the TETRAS activities of daily living (ADL) subscale and with 2 quality of life (QoL) scales.

**Methods:**

This noninterventional, cross-sectional, point-in-time survey of neurologists(n = 60), primary care physicians (n = 38), and their patients with ET (n = 1,003) used real-world data collected through the Adelphi ET Disease Specific Programme™. Physician-reported measures (TETRAS Performance Item 4 and TETRAS ADL total) and patient-reported QoL measures (generic EuroQol-5 Dimension 5 Level [EQ-5D-5 L] and ET-specific Quality of Life in Essential Tremor Questionnaire (QUEST)) were assessed with bivariate and multivariable analyses. Sensitivity analyses were also conducted.

**Results:**

The bivariate association between TETRAS Performance Item 4 score and TETRAS ADL total score was high (Pearson r = 0.761, *P* < 0.001). The bivariate associations between TETRAS Performance Item 4 score and EQ-5D-5 L index score (r = –0.410, *P* < 0.001) and between TETRAS ADL total score and EQ-5D-5 L index score (r = –0.543, *P* < 0.001) were moderate. The bivariate associations between TETRAS Performance Item 4 score and QUEST total score (r = 0.457, *P* < 0.001), and between TETRAS ADL total score and QUEST total score (r = 0.630, *P* < 0.001) were also moderate. These associations were unaltered by the inclusion of covariates.

**Discussion:**

This study showed that greater tremor severity (TETRAS Performance Item 4) was positively correlated with ADL impairment (TETRAS ADL) and negatively associated with QoL (EQ-5D-5 L and QUEST). TETRAS Performance Item 4 score is a robust predictor of TETRAS ADL total score, and TETRAS Performance Item 4 and TETRAS ADL total scores were robust predictors of the 2 QoL scales. The results demonstrate the value of TETRAS scores as valid endpoints for future clinical trials.

**Highlights:**

This real-world study assessed TETRAS scores as predictors of impaired QoL in ET. TETRAS Performance Item 4 and ADL were associated with EQ-5D-5 L and QUEST. TETRAS scores may serve as valid endpoints for future clinical trials.

## 1. Introduction

Essential tremor (ET) is among the most common movement disorders in adults. Patients with ET have upper limb action tremor and may commonly exhibit tremor in the head and voice, as well as other bodily regions over time [1,2].

An estimated 6.8 million adults are affected by ET in the United States (US), with higher prevalence in older age groups [3]. Common comorbidities among patients with ET include essential hypertension, pain disorders, lipid metabolism disorders, mood and anxiety disorders, and movement disorders such as dystonia, parkinsonism, and ataxia [2,4]. These can complicate disease management and pose significant clinical and public health challenges [4]. Current pharmacological interventions for ET have limited efficacy, and approximately 50% of treated patients may not respond optimally to oral standard-of-care, including propranolol and primidone [5,6,7,8].

ET is not fatal, but it progresses over time and impairs activities of daily living (ADL) [9,10,11,12]. Consequently, ET can negatively impact psychological well-being, overall quality of life (QoL), and ability to function independently [9,11,12,13]. The disease imposes a significant economic burden on both households and healthcare systems, affecting their financial capacity, productivity, and resource allocation [4,9,14].

The Essential Tremor Rating Assessment Scale (TETRAS) is a physician-administered, comprehensive clinical measurement tool consisting of a Performance subscale and an ADL subscale, designed for clinical trials of ET and for routine clinical assessment of treatment effects and disease progression [15]. The TETRAS Performance subscale assesses tremor amplitude. Upper limb tremor is present in all cases of ET and is usually the dominant symptom [13,16]. Item 4 of the TETRAS Performance subscale measures upper limb tremor and includes 3 maneuvers for each arm that assess postural and kinetic tremor [15]. TETRAS Performance Item 4 is known to be a reliable and sensitive measure of tremor severity [17]. The TETRAS ADL subscale assesses the impact of tremor on speech, upper limb function, and social activities. The Food and Drug Administration (FDA) has a preference for assessing ADL as a primary outcome in pivotal clinical trials, especially when the ADL scale is based on patient-reported outcomes [18].

The correlation between TETRAS Performance total score and TETRAS ADL total score is well established [15]. Item 4 and TETRAS ADL are being employed in clinical trials for ET as primary/secondary endpoints for regulatory decision making [19,20,21], but their relationships with each other and with QoL have not been formally assessed. Therefore, we examined the relationships among TETRAS Performance Item 4, TETRAS ADL total score, and QoL (assessed via the ET-specific Quality of Life in Essential Tremor Questionnaire [QUEST] and the generic patient-reported measure of health, EuroQol-5 Dimension 5 Level [EQ-5D-5 L]) through bivariate (i.e., unadjusted) and multivariable (i.e., adjusted) statistical analyses, using a real-world dataset. Of particular interest was the relative degree to which Item 4 and TETRAS ADL predicted QoL in a large outpatient cohort. Findings will further demonstrate the impact of ET on affected patients and facilitate clinical decision making as well as drug development by aiding in the evaluation of the potential benefits of new ET therapies.

## 2. Methods

### 2.1 Data source and patient population

The current study is a noninterventional, cross-sectional, point-in-time survey of primary care physicians (PCPs), neurologists, and their patients with ET. The study used real-world data collected in the US from March 2021 to August 2021 through an Adelphi ET Disease Specific Programme (DSP)™, using published standardized methodology [22]. Briefly, DSPs are large, multinational, observational studies of real-world clinical practice. Here, the data are reported by clinicians via online surveys and detailed patient records (including clinical decision-making questions) and by patients via self-reported questionnaires (including validated disease-specific patient-reported outcomes), which can inform current symptom prevalence and severity, and associated treatment practices for a range of common chronic disease areas [22,23]. In comparison to other data sources, the DSP is a unique dataset that collects clinical information reported by treating physicians, information on physician and patient attitudes to treatment, and patient-reported outcomes [22]. This study was approved by the Western Institutional Review Board (protocol number AG-8947).

The Adelphi ET dataset contains de-identified survey data from 1,003 patients diagnosed with ET, treated by 98 physicians (38 PCPs and 60 neurologists) (**Supplementary Figure 1**). All patients in the Adelphi ET dataset had physician-reported scores for primary and secondary endpoints (TETRAS measures). Physicians involved in the management of patients with ET in the US were identified from public lists of healthcare professionals and included in the study if they were current practicing physicians who treated 10 or more patients with diagnosed ET in a typical month. Each eligible and consenting physician provided information on their next 10 consecutive patients with ET, regardless of the reason for the visit. Patients with ET were ≥18 years old and not currently involved in a clinical trial.

A subset of 476 patients from the ET dataset completed the patient-reported surveys, of which 463 patients completed the EQ-5D-5 L and 456 patients completed the QUEST QoL assessments. These patients provided informed consent via a checkbox for use of their anonymized and aggregated data for research and publication in scientific journals. Patients had the right to opt out of the study at any time.

### 2.2 Study measures

#### 2.2.1 Patient characteristics

Patient demographics and clinical characteristics were obtained from the physicians and the patient-reported outcome tools for QoL assessment.

#### 2.2.2 Physician-reported measures

ET-related upper limb tremor was assessed using the TETRAS Performance subscale. ET-related ADL impairment was assessed using the the TETRAS AD subscale.

##### TETRAS Performance and ADL

TETRAS has Performance and ADL subscales, both highlighting upper limb tremor [15]. TETRAS Performance subscale contains 9 items and addresses head tremor (Item 1), face tremor (Item 2), voice tremor (Item 3), upper limb postural (forward horizontal extension and lateral extension with elbow flexed [wing posture]) and kinetic (finger-nose or chin-finger maneuvers) tremor (Item 4), lower limb tremor (Item 5), spiral drawing (Item 6), handwriting (Item 7), dot approximation (Item 8), and standing (Item 9). Each item is scored 0–4, in 0.5-point increments. Item 4 is the sum of 3 maneuvers (postural forward, wing posture, and kinetic) for each upper limb, resulting in an Item 4 score of 0–24. The Item 5 lower limb score (0–4) is the maximum postural (leg extended horizontally) or kinetic (heel-shin test) tremor in the right and left lower limbs. TETRAS Performance total score is calculated as the sum of scores from TETRAS Performance Items 1 through 9, with a range of 0 to 64 for each patient. TETRAS performance ratings have a logarithmic relationship with tremor amplitude, measured with a motion transducer [15,24,25]. Higher TETRAS Performance scores indicate increased tremor severity.

TETRAS ADL subscale contains 12 items that address speech impairment (Item 1), occupational impairment (Item 10), social impact (Item 12), and impairment in activities affected by upper limb tremor (remaining 9 items), with each item scored 0–4. TETRAS ADL total score is calculated as the sum of scores from Items 1 to 12, ranging from 0 to 48 [15]. Higher TETRAS ADL scores indicate higher impact of tremor on typical daily activities.

Cronbach’s alpha (α) statistics were computed to assess the internal consistency of TETRAS ratings by the physicians in this study. The computed α was 0.960 (95% confidence interval [CI]: 0.956–0.964) for TETRAS Performance subscale, 0.961 (95% CI: 0.955–0.966) for TETRAS Performance Item 4 score, and 0.948 (95% CI: 0.942–0.953) for TETRAS ADL subscale.

#### 2.2.3 Patient-reported measures

Patient QoL was assessed based on the patient-reported measures of EQ-5D-5 L and QUEST.

##### EQ-5D-5 L index score

The EQ-5D-5 L is a disease-agnostic measure of health status that compiles patient-reported assessments of 5 dimensions (i.e., mobility, self-care, usual activities, pain/discomfort, and anxiety/depression), with 5 possible response levels for each dimension (1 = no problem, 2 = slight problem, 3 = moderate problem, 4 = severe problem, 5 = unable to function) [26,27]. Responses to these items are converted into a single summary EQ-5D-5 L index score ranging from negative values (e.g., –0.573 to 1) [26], with 0 representing death, 1 representing perfect health, and negative values indicating a state worse than death [27]. The index score is obtained by deducting the appropriate weights from the value for full health. The weights for all possible EQ-5D health states are referred to as a “value set” [27]. The EQ-5D-5 L value set is available for the US and was derived from interviews in the US adult population, using the international standardized protocol developed by the EuroQoL group [26]. As the data for this study was collected in the US, the US EQ-5D-5 L value set was used.

##### QUEST

QUEST is a 30-item scale that measures QoL in patients with ET [28]. It has 5 subdomains: physical (9 items), psychosocial (9 items), communication (3 items), hobbies/leisure (3 items), and work/finance (6 items). Each subdomain score ranges from 0 to 100, with a higher score indicating greater dissatisfaction (additional details in **Supplementary Materials**). A total score is computed by calculating the mean of the 5 subdomain scores.

### 2.4 Statistical methods

Descriptive summary statistics were used to describe patient demographic and clinical characteristics, current comorbidities, treatment patterns, and ET-related characteristics. Bivariate analyses were conducted to assess the relationship between key dependent and independent variables. For variables with continuous data, Pearson correlation tests were conducted to measure the strength of the linear relationship with primary outcome variables. For variables with binary categorical data, Welch two-sample t-tests were conducted to test the null hypothesis that primary outcome means were equal across categories. For variables with multiple categories, an analysis of variance test was conducted to test the null hypothesis. Bivariate analyses included visual assessments and two-way scatter plots to assess the relationship between variables.

Multivariable core regression models were developed to assess the relationship between key study outcomes, controlling for key patient characteristics (i.e., age, depression/anxiety) impacting ET-related ADL impairment and QoL. Ordinary least squares (OLS) regression models were developed for bivariate analyses and core models. Potential covariates were considered for regression models if they exhibited a meaningful, statistically significant relationship with TETRAS ADL scores in bivariate analyses or had a theoretical basis for inclusion based on clinician inputs (provided by Drs. LCS and RJE). Patient-level covariates with the potential to independently impact ADL or QoL identified for core regression models included age, an indicator for current diagnosis of depression, an indicator for current diagnosis of anxiety, an indicator for current diagnosis of comorbidity potentially affecting movement/balance, Charlson Comorbidity Index (CCI) and body mass index (BMI). To ensure robustness of results, expansive multivariable models, in addition to the core model, with a broader set of covariates (based on clinician input) and alternative model specifications were also estimated in sensitivity analyses that incorporated additional covariates including sex, treatment status, alcohol use to alleviate tremor symptoms, BMI, CCI, household income (patient-reported), and an indicator for moderate to severe treatment-related side effects (patient-reported) (details in **Supplementary Materials**).

Multivariable OLS models included physician-level fixed effects (FE) to account for potential errors specific to individual physicians and clinical covariates (e.g., treatment status, TETRAS assessments, etc.). OLS with physician-level FE was used for regression models where TETRAS ADL total score was the dependent variable and TETRAS Performance Item 4 score or TETRAS Performance total score were the independent variables of interest. Analogous models with physician-level random effects (RE) were also estimated as sensitivity analyses.

Bivariate analyses were conducted in R (version 4.2), while all regression analyses were conducted in Stata/MP version 15 software (College Station, TX, USA). All data were visualized and reported using UNICOM^®^ Intelligence Reporter Version 7.5 or later (UNICOM Systems, Inc., Mission Hills, CA, USA). Statistical significance was set at *P* < 0.05.

## 3. Results

### 3.1 Patient characteristics

The demographic and clinical characteristics of the study population are summarized in Table 1 and **Supplementary Table 1**. In the overall population, the mean age was 64.9 ± 13.4 years, with 42.8% of patients younger than 65 years. Nearly half of the patients were female (47.5%), and a majority identified as White/Caucasian (75.3%), followed by African American (11.7%) and Asian (4.8%). Patients most commonly reported onset of ET symptoms in their 50s or 60s. Mean CCI score was 0.7 ± 1.2, with hypertension (49.4%), hyperlipidemia (29.4%), anxiety (24.3%), and depression (14.7%) being the most common comorbidities. Most patients (83.5%) were on prescription drugs for ET, with propranolol and primidone being the most common (39.0% and 30.9%, respectively). In the overall population, the mean (standard deviation [SD]) TETRAS Performance Item 4 and TETRAS ADL total scores were 9.3 (4.7) and 16.3 (9.5), respectively. Demographic and clinical characteristics of the subset of patients with EQ-5D-5 L and QUEST data were similar to those of the overall population.

**Table 1:** Demographic and clinical characteristics of the study population.


CHARACTERISTIC	ALL PATIENTS (*N* = 1,003)^1^	ALL PATIENTS WITH NONMISSING EQ-5D-5 L INDEX SCORES (*N* = 463)^2^	PATIENTS WITH NONMISSING QUEST TOTAL SCORES (*N* = 456)^3^

Female sex, % (*n*)^4^	47.5 (476)	47.3 (219)	48 (219)

Age (years), mean (SD)^4^	64.9 (13.4)	64.5 (13.4)	64.3 (13.4)

Age category, % (*n*)^4^			

0–18 years	0.1 (1)	0.2 (1)	0.2 (1)

19–64 years	42.7 (428)	45.6 (211)	46.5 (212)

64+ years	57.2 (574)	54.2 (251)	53.3 (243)

Race/ethnicity, % (*n*)			

White/Caucasian	75.3 (755)	79.9 (370)	79.8 (364)

Asian	4.8 (48)	2.8 (13)	2.6 (12)

African American	11.7 (117)	9.7 (45)	9.9 (45)

Hispanic	3.7 (37)	3.9 (18)	3.9 (18)

Other	4.6 (46)	3.7 (17)	3.7 (17)

**ET history**			

Start of ET symptoms, % (*n*)^5^			

Childhood	2.1 (21)	2.2 (10)	2.2 (10)

20’s	4.1 (41)	3.7 (17)	3.7 (17)

30’s	5.6 (56)	4.1 (19)	4.2 (19)

40’s	12.6 (126)	13.0 (60)	12.9 (59)

50’s	23.9 (240)	30.0 (139)	30.9 (141)

60’s	29.6 (297)	30.0 (139)	29.4 (134)

70’s	11.7 (117)	13.0 (60)	12.7 (58)

80’s	2.1 (21)	2.8 (13)	2.9 (13)

Unknown	8.4 (84)	1.3 (6)	1.1 (5)

**Comorbidities**			

CCI score, mean (SD)^6^	0.7 (1.2)	0.7 (1.3)	0.7 (1.3)

Comorbidities, % (*n*)			

Hypertension	49.4 (495)	49.2 (228)	48.7 (222)

Anxiety	24.3 (244)	31.3 (145)	31.6 (144)

Depression	14.7 (147)	17.1 (79)	17.5 (80)

Hyperlipidemia	29.4 (295)	30.2 (140)	30.7 (140)

Other^7^	43.4 (435)	41.7 (193)	40.8 (186)

None of the above	26.4 (265)	26.3 (122)	26.8 (122)

**ET treatment/procedures**			

ET treatment status/history, % (*n*)			

Currently prescribed a drug for treating ET	83.5 (838)	84.4 (391)	84.6 (386)

Propranolol	39.0 (391)	36.9 (171)	36.8 (168)

Primidone	30.9 (310)	31.3 (145)	31.4 (143)

Atenolol	7.3 (73)	11.0 (51)	11.2 (51)

Alprazolam	5.4 (54)	7.1 (33)	7.2 (33)

Clonazepam	7.4 (74)	6.3 (29)	5.9 (27)

Gabapentin	5.9 (59)	5.6 (26)	5.7 (26)

Topiramate	7.4 (74)	7.8 (36)	8.1 (37)

Other^8^	12.3 (123)	14.3 (66)	14.3 (65)

Past ET-related procedures, % (*n*)			

Deep-brain stimulation	6.5 (65)	7.8 (36)	7.9 (36)

Thalamotomy	3.4 (34)	3.7 (17)	3.7 (17)

Magnetic resonance-guided focused ultrasound	4.4 (44)	4.3 (20)	4.4 (20)

Other	2.5 (25)	3.0 (14)	3.1 (14)

None	77.4 (776)	78.2 (362)	77.9 (355)

Unknown	13.2 (132)	10.6 (49)	10.7 (49)

**TETRAS scores**			

TETRAS Performance total score, mean (SD)	21.4 (11.7)	22.9 (11.5)	22.9 (11.5)

TETRAS Performance Item 4 score, mean (SD)	9.3 (4.7)	9.9 (4.6)	9.8 (4.7)

TETRAS ADL total score, mean (SD)	16.3 (9.5)	17.4 (9.6)	17.4 (9.6)

**QoL scores**			

EQ-5D-5 L index^9^, mean (SD)	—	0.74 (0.21)	0.74 (0.21)

QUEST total score^9^, mean (SD)	—	24.6 (19.1)	24.6 (19.2)

QUEST subdomain scores^9^, mean (SD)			

QUEST communication	—	17.7 (23.8)	17.7 (23.9)

QUEST work finances	—	14.6 (21.4)	14.6 (21.5)

QUEST hobbies and leisure	—	26.4 (32.3)	26.3 (32.3)

QUEST physical	—	36.4 (22.8)	36.5 (22.9)

QUEST psychosocial	—	27.6 (21.3)	27.6 (21.5)


1. Table reports patient characteristics for all patients with data derived from standardized clinician reports in the Adelphi ET DSP™ *(N* = 1,003). 2. Table reports patient characteristics for all patients with data derived from patient-reported surveys in the Adelphi ET DSP™ with a nonmissing EQ-5D-5 L index score (*N* = 463). 3. Table reports patient characteristics for all patients with data derived from patient-reported surveys in the Adelphi ET DSP^TM with a nonmissing QUEST total score^ (*N* = 456). 4. In instances where responses differed between the standardized clinician reports and patient-reported surveys, responses from patient-reported questionnaires were utilized. 5. Age range of ET symptom onset was obtained from standardized clinician reports. Missing/unknown values were imputed using patient-reported age of onset from patient-reported surveys, where possible. Remaining missing values were assigned to “Unknown”. 6. Charlson Comorbidity Index calculated using standard CCI comorbidity weights [29]. 7. Other comorbidities included: myocardial infarction, congestive heart failure, peripheral vascular disease, cerebrovascular disease, hemiplegia or paraplegia, dementia, chronic pulmonary disease, rheumatologic disease, peptic ulcer disease, diabetes without chronic complications, diabetes with chronic complications, renal disease, any malignancy including leukemia and lymphoma, metastatic solid tumor, mild liver disease, moderate or severe liver disease, AIDS/HIV, hypotension, migraine headaches, alcohol dependency, cannabis/cannabinoid dependency, and Parkinson’s disease. 8. Other currently prescribed ET treatments included (<5% in all cohorts): sotalol, nadolol, lorazepam, diazepam, pregabalin, zonisamide, clozapine, nimodipine, and botulinum toxin. 9. Missing values were removed from both the numerator and denominator when computing QUEST subdomain scores; QUEST total score was calculated as the unweighted average across all QUEST subdomain scores. ADL, activities of daily living; AIDS, acquired immunodeficiency syndrome; CCI, Charlson comorbidity index; DSP, Disease Specific Programme; EQ-5D-5 L, EuroQol-5 Dimension 5 Level; ET, essential tremor; HIV, human immunodeficiency virus; QUEST, Quality of Life in Essential Tremor Questionnaire; TETRAS, The Essential Tremor Rating Assessment Scale; SD, standard deviation.

### 3.2 Association between upper limb tremor and ADL

**Figure 1:**
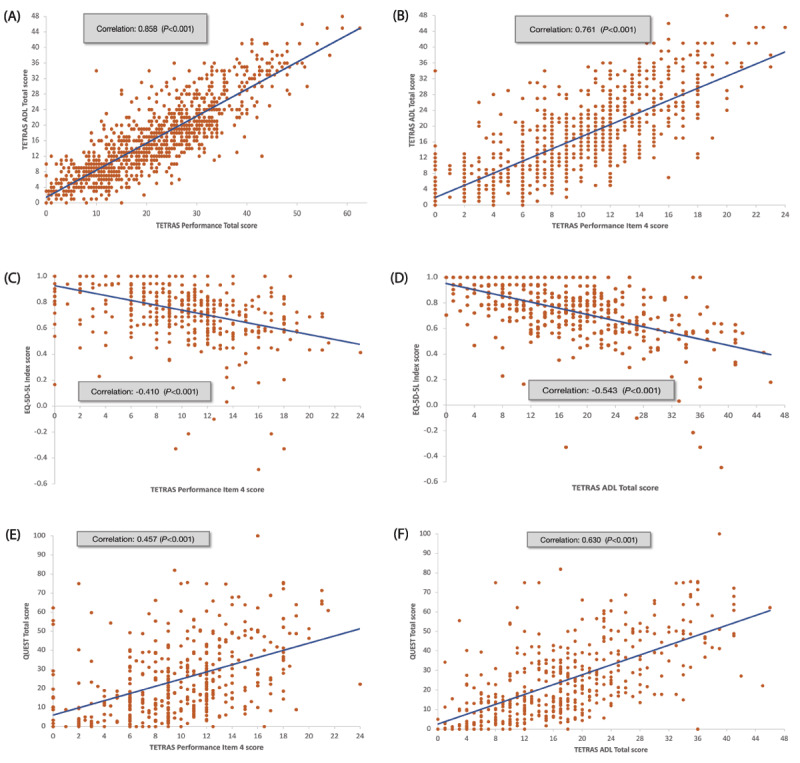
Correlation analysis results. (A) TETRAS ADL total score with TETRAS Performance total score (B) TETRAS ADL total score with TETRAS Performance Item 4 score (C) EQ-5D-5 L index score and TETRAS Performance Item 4 score (D) EQ-5D-5 L index score and TETRAS ADL total score (E) QUEST total score and TETRAS Performance Item 4 score (F) QUEST total score and TETRAS ADL total score ADL, activities of daily living; EQ-5D-5 L, EuroQol-5 Dimension 5 Level; QUEST, Quality of Life in Essential Tremor Questionnaire; TETRAS, The Essential Tremor Rating Assessment Scale.

There was a high correlation between TETRAS Performance total score and TETRAS ADL total score in the bivariate analysis (unadjusted OLS coefficient = 0.70, Pearson r = 0.858, *P* < 0.001) (Figure 1A), as was the association between TETRAS Performance Item 4 score and TETRAS ADL total score (unadjusted OLS coefficient = 1.54, Pearson r = 0.761, *P* < 0.001) (Figure 1B and **Supplementary Table 2**).

Results from the multivariable analyses are shown in Table 2. After adjusting for other factors (age; an indicator for current diagnoses of depression, anxiety; comorbidity potentially affecting movement/balance), the mean TETRAS ADL total score increased by 1.420 points for every 1-point increase in TETRAS Performance Item 4 score, suggesting a strong association between ET-related upper limb tremor and ADL impairment (*P* < 0.001). The covariates of age (*P* < 0.001), depression (*P* = 0.003), and comorbidity affecting balance/movement (*P* < 0.001) were significantly associated with increased ADL total score. Similar results were observed for the expanded model in sensitivity analyses (**Supplementary Table 3**).

**Table 2:** Core multivariable model results for the associations of TETRAS Performance with ADL, and TETRAS Performance Item 4 and ADL with EQ-5D-5 L index score and QUEST total score.


VARIABLE	COEFFICIENT (ROBUST SE)	t	*P* VALUE

**TETRAS Performance total score vs. TETRAS ADL total score** ^1^			

TETRAS Performance total score	0.68*** (0.021)	32.878	<0.001

Age	0.04*** (0.015)	2.913	0.004

Anxiety	–0.12 (0.352)	–0.331	0.741

Depression	1.57*** (0.590)	2.661	0.009

Balance/movement comorbidity	1.33** (0.526)	2.529	0.013

Constant	–1.33 (0.960)	–1.390	0.168

**TETRAS Performance Item 4 score vs. TETRAS ADL total score** ^2^			

TETRAS Performance Item 4 score	1.42*** (0.070)	20.356	<0.001

Age	0.09*** (0.019)	4.694	<0.001

Anxiety	–0.13 (0.422)	–0.307	0.759

Depression	2.16*** (0.695)	3.104	0.003

Balance/movement comorbidity	2.53*** (0.642)	3.940	<0.001

Constant	–3.14** (1.200)	–2.615	0.010

**TETRAS Performance Item 4 score vs. EQ-5D-5 L index score** ^3^			

TETRAS Performance Item 4 score	–0.0148*** (0.002)	–6.023	<0.001

Age	–0.0032*** (0.001)	–3.788	<0.001

Anxiety	–0.0456** (0.021)	–2.161	0.034

Depression	–0.0942*** (0.032)	–2.912	0.005

CCI	–0.0093 (0.008)	–1.218	0.228

BMI	–0.0019 (0.002)	–0.838	0.405

Constant	1.1787*** (0.081)	14.578	<0.001

**TETRAS ADL total score vs. EQ-5D-5 L index score** ^4^			

TETRAS ADL total score	–0.0100*** (0.001)	–7.171	<0.001

Age	–0.0025*** (0.001)	–3.148	0.003

Anxiety	–0.0370 (0.020)	–1.819	0.074

Depression	–0.0693*** (0.026)	–2.657	0.010

CCI	–0.0031 (0.007)	–0.436	0.664

BMI	–0.0026 (0.002)	–1.104	0.274

Constant	1.1743*** (0.080)	14.631	<0.001

**TETRAS Performance Item 4 score vs. QUEST total score** ^5^			

TETRAS Performance Item 4 score	1.720*** (0.23)	7.439	<0.001

Age	–0.002 (0.08)	–0.030	0.977

Anxiety	3.305 (2.33)	1.419	0.161

Depression	11.581*** (2.93)	3.959	<0.001

CCI	0.863 (0.61)	1.405	0.165

BMI	–0.213 (0.19)	–1.102	0.275

Constant	9.833 (8.25)	1.192	0.238

**TETRAS ADL total score vs. QUEST total score** ^6^			

TETRAS ADL total score	1.201*** (0.09)	13.838	<0.001

Age	–0.081 (0.07)	–1.100	0.275

Anxiety	2.231 (1.97)	1.130	0.263

Depression	8.566*** (2.44)	3.511	0.001

CCI	0.130 (0.53)	0.243	0.809

BMI	–0.152 (0.19)	–0.810	0.421

Constant	10.664 (7.96)	1.339	0.185


1. Number of observations = 1,003, number of physicians = 98, overall R-squared value = 0.750, F-value = 262.9. 2. Number of observations = 1,003, number of physicians = 98, overall R-squared value = 0.613, F-value = 120.2. 3. Number of observations = 463, overall R-squared value = 0.275, F-value = 19.18. 4. Number of observations = 463, overall R-squared value = 0.353, F-value = 28.66. 5. Number of observations = 456, overall R-squared value = 0.291, F-value = 22.98. 6. Number of observations = 456, overall R-squared value = 0.438, F-value = 54.16. ***P < 0.01, **P < 0.05. ADL, activities of daily living; BMI, body mass index; CCI, Charlson comorbidity index; EQ-5D-5 L, EuroQol-5 Dimension 5 Level; QUEST, Quality of Life in Essential Tremor Questionnaire; SE, standard error; TETRAS, The Essential Tremor Rating Assessment Scale.

### 3.3 Associations of upper limb tremor and ADL with QoL

#### 3.3.1 Upper limb tremor and generic QoL

A moderate association was observed between TETRAS Performance Item 4 score and the generic QoL EQ-5D-5 L index score in the bivariate analysis (unadjusted OLS coefficient = –0.019, Pearson r = –0.410, *P* < 0.001) (Figure 1C and **Supplementary Table 4**).

Results from the multivariable analysis are shown in Table 2. After adjusting for other factors (age, an indicator for current diagnoses of depression or anxiety, CCI, and BMI), the mean EQ-5D-5 L index score decreased by 0.0148 points for every 1-point increase in TETRAS Performance Item 4 score (*P* < 0.001). The covariates of age (*P* < 0.001), anxiety (*P* = 0.034), and depression (*P* = 0.005) were significantly associated with a decreased EQ-5D-5 L index score. Similar results were observed for the expanded model in sensitivity analyses (**Supplementary Table 5**).

#### 3.3.2 ADL and generic QoL

There was a moderate correlation between TETRAS ADL total score and the EQ-5D-5 L index score in the bivariate analysis (unadjusted OLS coefficient = –0.012, Pearson r = –0.543, *P* < 0.001) (Figure 1D and **Supplementary Table 4**).

Results from the multivariable analysis are shown inTable 2. After adjusting for other factors (age, an indicator for current diagnoses of depression or anxiety, CCI, and BMI), the mean EQ-5D-5 L index score decreased by 0.0100 points for every 1-point increase in TETRAS ADL total score (*P* < 0.001). The covariates of age (*P* = 0.003) and depression (*P* = 0.010) were significantly associated with decreased EQ-5D-5 L index score. Similar results were observed for the expanded model in sensitivity analyses (**Supplementary Table 6**).

#### 3.3.3 Upper limb tremor and ET-specific QoL

A moderate association was observed between TETRAS Performance Item 4 score and QUEST total score in the bivariate analysis (unadjusted OLS coefficient = 1.886, Pearson r = 0.457, *P* < 0.001) (Figure 1E and **Supplementary Table 4**).

Results from the multivariable analysis are shown in Table 2. After adjusting for other factors (age, an indicator for current diagnoses of depression or anxiety, CCI, and BMI), the mean QUEST total score increased by 1.720 points for every 1-point increase in TETRAS Performance Item 4 score (*P* < 0.001). The covariate of depression was significantly associated with an increased QUEST Total score (*P* < 0.001). Similar results were observed for the expanded model in sensitivity analyses (**Supplementary Table 7**).

Multivariable analysis estimates based on results from the core regression model evaluating the association between TETRAS Performance Item 4 and QUEST subdomain scores are shown in Table 3. QUEST Communication (*P* < 0.05), Work and Finances, Hobbies and Leisure, Physical, and Psychosocial (all others *P* < 0.01) subdomain scores were significantly associated with TETRAS Item 4 score.

**Table 3:** Core multivariable model results for the associations of TETRAS Performance Item 4 and ADL with QUEST subdomain scores.


VARIABLE	QUEST COMMUNICATION	QUEST WORK AND FINANCES	QUEST HOBBIES AND LEISURE	QUEST PHYSICAL	QUEST PSYCHOSOCIAL

**TETRAS Performance Item 4 score vs. QUEST subdomain scores** ^1^ **, coefficient (SE)**					

TETRAS Performance Item 4 score	0.9484** (0.448)	1.5074*** (0.236)	2.1021*** (0.345)	2.3597*** (0.263)	1.6730*** (0.222)

Age	0.1101 (0.108)	–0.2565*** (0.085)	0.0625 (0.126)	0.1707** (0.075)	–0.0963 (0.095)

Anxiety	3.7376 (3.237)	0.4346 (2.703)	2.3045 (4.125)	3.5915 (2.577)	6.3466** (2.478)

Depression	10.4082** (4.990)	7.0642 (3.727)	14.1426*** (4.890)	9.1765*** (3.141)	17.0781*** (2.988)

CCI	2.0896 (1.096)	1.0235 (0.777)	0.6555 (1.232)	0.3964 (0.764)	0.2374 (0.780)

BMI	–0.3373 (0.243)	–0.2012 (0.239)	–0.0245 (0.378)	–0.2958 (0.238)	–0.2178 (0.179)

Constant	5.9706 (10.418)	19.6389** (9.706)	–1.3226 (12.692)	7.1582 (8.992)	18.0999** (8.791)

**TETRAS ADL total score vs. QUEST subdomain scores** ^2^ **, coefficient (SE)**					

TETRAS ADL total score	1.1064*** (0.194)	0.9492*** (0.123)	1.3433*** (0.152)	1.4053*** (0.105)	1.1577*** (0.090)

Age	–0.0134 (0.101)	–0.3084*** (0.088)	–0.0122 (0.126)	0.1018 (0.076)	–0.1737 (0.094)

Anxiety	1.9160 (2.744)	–0.2149 (2.566)	1.2855 (3.910)	2.7949 (2.623)	5.3282** (2.024)

Depression	7.5100 (4.369)	4.7090 (3.671)	10.7777** (4.684)	5.7048 (3.217)	14.1675*** (2.553)

CCI	1.3760 (0.990)	0.4572 (0.768)	–0.1141 (1.185)	–0.4228 (0.697)	–0.4565 (0.715)

BMI	–0.3611 (0.225)	–0.1337 (0.253)	0.0663 (0.360)	–0.1767 (0.224)	–0.1553 (0.190)

Constant	6.1928 (10.208)	20.4646** (9.799)	–0.1991 (11.953)	8.5432 (8.801)	19.0037** (8.828)


1. QUEST Communication: Number of observations = 459, overall R-squared value = 0.126, F-value = 9.58; QUEST Work and Finances: Number of observations = 458, overall R-squared value = 0.134, F-value = 10.48; QUEST Hobbies and Leisure: Number of observations = 458, overall R-squared value = 0.150, F-value = 12.24; QUEST Physical: Number of observations = 460, overall R-squared value = 0.329, F-value = 26.73; QUEST Psychosocial: Number of observations = 460, overall R-squared value = 0.310, F-value = 24.43. 2. QUEST Communication: Number of observations = 459, overall R-squared value = 0.262, F-value = 17.99; QUEST Work and Finances: Number of observations = 458, overall R-squared value = 0.189, F-value = 12.82; QUEST Hobbies and Leisure: Number of observations = 458, overall R-squared value = 0.202, F-value = 21.08; QUEST Physical: Number of observations = 460, overall R-squared value = 0.415, F-value = 68.89; QUEST Psychosocial: Number of observations = 460, overall R-squared value = 0.418, F-value = 43.09. ***P < 0.01, **P < 0.05. ADL, activities of daily living; BMI, body mass index; CCI, Charlson comorbidity index; QUEST, Quality of Life in Essential Tremor Questionnaire; SE, standard error; TETRAS, The Essential Tremor Rating Assessment Scale.

#### 3.3.4 ADL and ET-Specific QoL

There was a moderate correlation between TETRAS ADL total score and QUEST total score in the bivariate analysis (unadjusted OLS coefficient = 1.264, Pearson r = 0.630, *P* < 0.001) (Figure 1F and **Supplementary Table 4**).

Results from the multivariable analysis are shown in Table 2. After adjusting for other factors (age, an indicator for current diagnoses of depression or anxiety, CCI, and BMI), the mean QUEST total score increased by 1.201 points for every 1-point increase in TETRAS ADL total score (*P* < 0.001). The covariate of depression was significantly associated with an increased QUEST total score (*P <* 0.001). Similar results were observed for the expanded model in sensitivity analyses (**Supplementary Table 8**).

Multivariable analysis estimates based on results from the core regression model evaluating the association between TETRAS ADL total score and QUEST subdomain scores are shown in Table 3. These results demonstrate that the QUEST Communication, Work and Finances, Hobbies and Leisure, Physical, and Psychosocial subdomain scores were significantly associated with TETRAS ADL total score (*P* < 0.01).

## 4. Discussion

Our analyses of real-world data collected from physicians and patients with ET revealed that TETRAS Performance Item 4 score was a robust predictor of TETRAS ADL total score. TETRAS Performance Item 4 and TETRAS ADL total scores showed significant associations with generic and ET-specific QoL. These results indicate that TETRAS ADL and Performance Item 4 measure ET-related functional impairment and patient’s QoL, and show that greater tremor severity was positively correlated with ADL impairment and negatively associated with QoL.

The correlation between TETRAS ADL total score and TETRAS Performance total score was similar to a previous report (r = 0.887, *P* < 0.0001) [25]. This study also found a robust correlation between TETRAS Performance Item 4 score and ADL total score (r = 0.761, *P* < 0.001). The directional results and statistical estimates for TETRAS Performance total score were similar to those produced by the core multivariable regression model with TETRAS Performance Item 4 score as the independent variable. Overall, the model including TETRAS Performance total score explained more of the variance in ADL impairment scores (overall R-squared of 0.750 vs. 0.613 in core models), potentially due to TETRAS Performance total score being a more expansive measure of ET tremor (not limited strictly to upper limb tremor). Several patients in the dataset registered low TETRAS Performance Item 4 scores with higher levels of severity in other categories of TETRAS Performance.

Both ET-related upper limb tremor (TETRAS Performance Item 4 score) and ADL impairment (TETRAS ADL total score) were associated with reduced QoL assessed via the generic EQ-5D-5 L index score (*P* < 0.001). These results highlight significant associations between TETRAS Performance Item 4 score, ADL impairment score and QoL in patients with ET. Overall, the core model including TETRAS ADL total score explained more of the variance in EQ-5D-5 L scores compared with models including TETRAS Performance Item 4 score (overall R-squared of 0.353 vs. 0.275 in core models). This can be attributed to the fact that the TETRAS ADL total score directly captures the magnitude of impairment in everyday life and is, therefore, more proximal to the lived experience of patients with ET.

Both upper limb tremor (TETRAS Performance Item 4 score) and ADL impairment (TETRAS ADL total score) were associated with a reduced QoL assessed via QUEST total score (*P* < 0.001), an ET-specific QoL measure [28,30]. Similar to regressions in EQ-5D-5 L scores, the R-squared for the core model incorporating TETRAS Performance Item 4 score was lower compared to the model incorporating TETRAS ADL total score (0.291 vs. 0.438), potentially due to the close overlap between the TETRAS ADL and the QUEST patient questionnaire. The significant associations of TETRAS Performance Item 4 and ADL with QUEST subdomain scores indicate that TETRAS Performance Item 4 and TETRAS ADL scores are relevant to a broad range of patient-reported QoL domains. TETRAS ADL total score had a robust association with the physical subdomain of QUEST (Table 3), likely because the content of TETRAS ADL total score aligns closely with this subdomain and should contribute to 20% (1 of 5 subdomains) of the QUEST total score. Patient age was positively associated with mean QUEST Physical score but negatively associated with mean QUEST Work and Finances score, likely due to low (or inapplicable) work and finance-related impairment for retirees in the sample. As expected, diagnosed anxiety/depression had the largest impact on QUEST Psychosocial score for both regressions involving TETRAS Performance Item 4 and TETRAS ADL total scores.

The TETRAS Performance subscale Item 4 is used in several clinical trials evaluating the efficacy of pharmacotherapies for the treatment of ET [19,20,21] but is not well validated as an isolated measure of tremor severity. To identify the unmet self-perceived needs and treatment needs of patients with ET in the US, responses to a 6-item patient-centeredness questionnaire showed that 12.7% of the respondents (*N* = 1,418) felt the need for a more quantitative and objective way of assessing tremor and tracking progression [31]. The present study demonstrated that TETRAS Performance Item 4 scores are statistically associated with impairment in ADL and QoL. This suggests that improvements in the TETRAS Performance Item 4 and/or TETRAS ADL scores are likely to correspond to improvements in patient QoL, be meaningful to the patient experience, and should be considered in future clinical trials.

All associations reported in the present study were robust, as there were minimal changes in the magnitude of the associations during various sensitivity analyses. This suggests that the associations observed are unlikely to be influenced by confounding variables. For example, increased age can negatively affect both TETRAS ADL total score and QoL measures, but increased age did not appear to confound the relationship between the 2 variables.

This study had several strengths. The Adelphi ET DSP™ is a real-world, observational study of physicians and patients consulting for ET, reflecting the actual treatment and prescribing decisions made by physicians in clinical practice. This independent data source provides unbiased reporting, without any preconceived hypotheses at the design stage and includes all patients (insured and noninsured), ensuring comprehensive coverage. The dataset captures important variables, such as clinical characteristics, complete treatment history, and reasons for prescribing or stopping treatment, through standardized clinician reports. The study methodology aimed to reduce selection bias by collecting data from the next set of consecutively treated patients, making the sample representative of a typical practice population. To the authors’ knowledge, this study covers the largest sample of TETRAS reported for real-world patients with ET to date, and is one of the few studies reporting TETRAS scores outside a clinical trial setting.

This study has several limitations. There is still a possibility of selection bias due to a higher likelihood of inclusion of those who seek consultation more frequently. The sample may be less likely to include patients who are on stable doses of medication and not experiencing changes in their tremor. The inclusion of patients treated with surgery in the study population could potentially bias the overall data, if treatment-related issues and adverse events associated with the surgical treatment have a differential impact on QoL compared to patients not treated with surgery. The recruitment of physicians could have been influenced by their willingness to participate, as well as practical factors such as their geographical location. The diagnosis in the target patient group was based primarily on the judgment and diagnostic skill of the physician rather than on requiring the physician to apply formal ET diagnostic criteria. However, all patients included in this study had a physician-confirmed diagnosis of ET, a requirement for patient eligibility stipulated in the study materials provided to participating physicians. To ensure ET was not misclassified in this study (e.g., as Parkinson’s disease), physicians made their clinical determination based on the ET criterion, 3 years of bilateral action tremor. While not every ET diagnosis was made by a neurologist, we found the overall TETRAS Performance scores mirror that of other clinic-based populations including the initial TETRAS validation study [15]. In addition, TETRAS Item 4 scores strongly reflect upper limb action tremor, which is a requirement for the diagnosis of ET. Further, the patient-level covariates for core regression models in this study were informed by clinical input, and included age and indicators for a current diagnosis of depression, anxiety, and a comorbidity potentially affecting movement or balance, such as Parkinson’s disease, cerebrovascular disease, hemiplegia/paraplegia, or dementia. The physicians in this study were not specifically trained on how to use TETRAS; nevertheless, the association observed between TETRAS Performance total score and TETRAS ADL total score was nearly identical to that observed in the original TETRAS validation study involving physicians who were specifically trained on TETRAS (r = 0.858 vs. r = 0.887) [25]. To account for some physician-to-physician variance due to lack of physician training and other unobserved or unmodeled factors related to physician or practice (experience, specialty, familiarity with ET), physician-level fixed effects were used.

The analysis of real-world data from physicians and their patients with ET underscores the importance of TETRAS scores as strong predictors of impaired QoL in patients with ET. It offers valuable insights into the interplay between ET-related upper limb tremor, functional limitations, and the impact of ET on patient well-being. Together, our results further support that improvements in TETRAS Performance Item 4 and TETRAS ADL scores can robustly capture patient experience and serve as valid endpoints in future clinical trials.

## Additional File

The additional file for this article can be found as follows:

10.5334/tohm.877.s1 Supplementary Materials. Supplementary methods, Supplementary results, Supplementary Tables and a Supplementary Figure.

## References

[1] Bhatia KP, Bain P, Bajaj N, Elble RJ, Hallett M, Louis ED, et al. Tremor Task Force of the International Parkinson and Movement Disorder Society. Consensus Statement on the classification of tremors. from the task force on tremor of the International Parkinson and Movement Disorder Society. Mov Disord. 2018; 33(1): 75–87. DOI: 10.1002/mds.27121 29193359 PMC6530552

[2] Lenka A, Jankovic J. Tremor syndromes: An updated review. Front Neurol. 2021; 12: 684835. Published 2021 Jul 26. DOI: 10.3389/fneur.2021.684835 34381412 PMC8350038

[3] Furtado J, Lally C, Flanders WD, Gerbasi ME, Maserejian N. Estimation of global age-specific prevalence of essential tremor by literature review of population-based studies. Presentation at International Conference on Pharmacoepidemiology & Therapeutic Risk Management, Halifax, Nova Scotia; 2023.

[4] Dai D, Samiian A, Fernandes J, Coetzer H. Multiple comorbidities, psychiatric disorders, healthcare resource utilization and costs among adults with essential tremor: A retrospective observational study in a large US commercially insured and Medicare Advantage population. J Health Econ Outcomes Res. 2022; 9(2): 37–46. Published 2022 Aug 15. DOI: 10.36469/001c.37307 36051002 PMC9378814

[5] Louis ED. Treatment of essential tremor: Are there issues we are overlooking? Front Neurol. 2012; 2: 91. Published 2012 Jan 13. DOI: 10.3389/fneur.2011.00091 22275907 PMC3257846

[6] Zesiewicz TA, Elble RJ, Louis ED, Gronseth GS, Ondo WG, Dewey RB Jr., et al. Evidence-based guideline update: treatment of essential tremor: Report of the Quality Standards subcommittee of the American Academy of Neurology. Neurology. 2011; 77(19): 1752–1755. DOI: 10.1212/WNL.0b013e318236f0fd 22013182 PMC3208950

[7] Koller WC, Vetere-Overfield B. Acute and chronic effects of propranolol and primidone in essential tremor. Neurology. 1989; 39(12): 1587–1588. DOI: 10.1212/wnl.39.12.1587 2586774

[8] Diaz NL, Louis ED. Survey of medication usage patterns among essential tremor patients: movement disorder specialists vs. general neurologists. Parkinsonism Relat Disord. 2010; 16(9): 604–607. DOI: 10.1016/j.parkreldis.2010.07.011 20691629 PMC2963696

[9] Gerbasi ME, Nambiar S, Reed S, Hennegan K, Hadker N, Eldar-Lissai A, et al. Essential tremor patients experience significant burden beyond tremor: A systematic literature review. Front Neurol. 2022; 13: 891446. Published 2022 Jul 22. DOI: 10.3389/fneur.2022.891446 35937052 PMC9354397

[10] Gupta HV, Pahwa R, Dowell P, Khosla S, Lyons KE. Exploring essential tremor: Results from a large online survey. Clin Park Relat Disord. 2021; 5: 100101. Published 2021 Jun 25. DOI: 10.1016/j.prdoa.2021.100101 34988425 PMC8710410

[11] Louis ED, Machado DG. Tremor-related quality of life: A comparison of essential tremor vs. Parkinson’s disease patients. Parkinsonism Relat Disord. 2015; 21(7): 729–735. DOI: 10.1016/j.parkreldis.2015.04.019 25952960 PMC4764063

[12] Morgan S, Kellner S, Gutierrez J, Collins K, Rohl B, Migliore F, et al. The experience of essential tremor caregivers: Burden and its correlates. Front Neurol. 2017; 8: 396. Published 2017 Aug 14. DOI: 10.3389/fneur.2017.00396 28855888 PMC5557742

[13] Louis ED. Essential tremor as a neuropsychiatric disorder. J Neurol Sci. 2010; 289(1–2): 144–148. DOI: 10.1016/j.jns.2009.08.029 19720384 PMC2813410

[14] Gerbasi ME, Pahwa R, Nunag D, Nejati M, Epstein AJ. Healthcare resource utilization and direct healthcare spending among Medicare patients with essential tremor. Presentation at AMCP Nexus, Orlando, FL. October 16–19, 2023.

[15] Elble RJ. The essential tremor rating assessment scale. J Neurol Neuromedicine. 2016; 1: 34–38. DOI: 10.29245/2572.942X/2016/4.103828405636

[16] Shanker V. Essential tremor: Diagnosis and management. BMJ. 2019; 366: l4485. Published 2019 Aug 5. DOI: 10.1136/bmj.l4485 31383632

[17] Ondo W, Hashem V, LeWitt PA, Pahwa R, Shih L, Tarsy D, et al. Comparison of the Fahn-Tolosa-Marin Clinical Rating Scale and the Essential Tremor Rating Assessment Scale. Mov Disord Clin Pract. 2017; 5(1): 60–65. Published 2017 Nov 23. DOI: 10.1002/mdc3.12560 30363460 PMC6174461

[18] FDA Draft Guidance. Patient-Focused Drug Development: Selecting, Developing, or Modifying Fit-for-Purpose Clinical Outcome Assessments. FDA.gov, June 2022. Available from: https://www.fda.gov/media/159500/download.

[19] A Clinical Trial of 2 Doses of PRAX-944 in Participants With Essential Tremor. ClinicalTrials.gov Identifier: NCT05021991. Updated March 23, 2023. Accessed Aug 29, 2023. https://classic.clinicaltrials.gov/ct2/show/NCT05021991.

[20] A Study To Assess the Safety and Efficacy of JZP385 in the Treatment of Adults With Moderate to Severe Essential Tremor (ET). ClinicalTrials.gov Identifier: NCT05122650. Updated Aug 28, 2023. Accessed Aug 29, 2023. https://classic.clinicaltrials.gov/ct2/show/NCT05122650.

[21] Study to Evaluate SAGE-324 in Participants With Essential Tremor. ClinicalTrials.gov Identifier: NCT05173012. Updated Aug 14, 2023. Accessed Aug 29, 2023. https://classic.clinicaltrials.gov/ct2/show/NCT05173012.

[22] Anderson P, Benford M, Harris N, Karavali M, Piercy J. Real-world physician and patient behaviour across countries: Disease-Specific Programmes – a means to understand. Curr Med Res Opin. 2008; 24(11): 3063–3072. DOI: 10.1185/03007990802457040 18826746

[23] Anderson P, Higgins V, de Courcy J, Doslikova K, Davis VA, Karavali M, et al. Real-world evidence generation from patients, their caregivers and physicians supporting clinical, regulatory and guideline decisions: an update on Disease Specific Programmes. Curr Med Res Opin. 2023; 39(12): 1707–1715. DOI: 10.1080/03007995.2023.2279679 37933204

[24] Elble RJ. Estimating change in tremor amplitude using clinical ratings: Recommendations for clinical trials. Tremor Other Hyperkinet Mov (N Y). 2018; 8: 600. Published 2018 Oct 11. DOI: 10.5334/tohm.455 PMC6802602 31637097

[25] Elble R, Comella C, Fahn S, Hallett M, Jankovic J, Juncos JL, et al. Reliability of a new scale for essential tremor. Mov Disord. 2012; 27(12): 1567–1569. DOI: 10.1002/mds.25162 23032792 PMC4157921

[26] Pickard AS, Law EH, Jiang R, Pullenayegum E, Shaw JW, Xie F, et al. United States valuation of EQ-5D-5 L health states using an international protocol. Value Health. 2019; 22(8): 931–941. DOI: 10.1016/j.jval.2019.02.009 31426935

[27] EuroQol Research Foundation. EQ-5D-5 L User Guide, 2019. Available from: https://euroqol.org/publications/user-guides.

[28] Tröster AI, Pahwa R, Fields JA, Tanner CM, Lyons KE. Quality of life in Essential Tremor Questionnaire (QUEST): Development and initial validation. Parkinsonism Relat Disord. 2005; 11(6): 367–373. DOI: 10.1016/j.parkreldis.2005.05.009 16103000

[29] Quan H, Li B, Couris CM, Fushimi K, Graham P, Hider P, et al. Updating and validating the Charlson comorbidity index and score for risk adjustment in hospital discharge abstracts using data from 6 countries. Am J Epidemiol. 2011; 173(6): 676–682. DOI: 10.1093/aje/kwq433. 21330339

[30] Elble R, Bain P, Forjaz MJ, Haubenberger D, Testa C, Goetz CG, et al. Task force report: scales for screening and evaluating tremor: critique and recommendations. Mov Disord. 2013; 28(13): 1793–1800. DOI: 10.1002/mds.25648 24038576

[31] Louis ED, Rohl B, Rice C. Defining the treatment gap: What essential tremor patients want that they are not getting. Tremor Other Hyperkinet Mov (N Y). 2015; 5: 331. Published 2015 Aug 14. DOI: 10.5334/tohm.239 26317044 PMC4548969

