# Supplementary material for: Associations Among Tremor Amplitude, Activities of Daily Living, and Quality of Life in Patients with Essential Tremor
Source: Tremor Other Hyperkinet Mov (N Y). 2024 May 3;14:22. doi: 10.5334/tohm.877 (PMC11067966; doi:10.5334/tohm.877)
Supplement: Supplementary Materials. — Supplementary methods, Supplementary results, Supplementary Tables and a Supplementary Figure. [file tohm-14-1-877-s1.pdf]

SUPPLEMENTARY MATERIALS

Supplementary methods

QUEST Subdomain scores

QUEST Communication Subdomain score

This score was calculated as the sum of non-missing scores from QUEST Item 1 through QUEST Item 3, divided by 4 times the number of non-missing responses from QUEST Item 1 through QUEST Item 3. The resulting score was then multiplied by 100, generating a subdomain score ranging from 0 to 100 for each patient.

QUEST Work and Finances Subdomain score

This score was calculated as the sum of non-missing scores from QUEST Item 4 through QUEST Item 9, divided by 4 times the number of non-missing responses from QUEST Item 4 through QUEST Item 9. The resulting score was then multiplied by 100, generating a subdomain score ranging from 0 to 100 for each patient.

QUEST Hobbies and Leisure Subdomain score

This score was calculated as the sum of non-missing scores from QUEST Item 10 through QUEST Item 12, divided by 4 times the number of non-missing responses from QUEST Item 10 through QUEST Item 12. The resulting score was then multiplied by 100, generating a subdomain score ranging from 0 to 100 for each patient.

QUEST Physical Subdomain Score

This score was calculated as the sum of non-missing scores from QUEST Item 13 through QUEST Item 21, divided by 4 times the number of non-missing responses from QUEST Item 13 through QUEST Item 21. The resulting score was then multiplied by 100, generating a subdomain score ranging from 0 to 100 for each patient.

QUEST Psychosocial Subdomain Score

This score was calculated as the sum of non-missing scores from QUEST Item 22 through QUEST Item 30, divided by 4 times the number of non-missing responses from QUEST Item 22 through QUEST Item 30. The resulting score was then multiplied by 100, generating a subdomain score ranging from 0 to 100 for each patient.

Core model specifications

Association between upper limb tremor and ADL impairment

For core regression models, four patient-level covariates identified based on clinician inputs included age, an indicator for current diagnosis of depression, an indicator for current diagnosis of anxiety, and an indicator for current diagnosis of comorbidity potentially affecting movement/balance (patients were assigned a “1” if they had a diagnosis of Parkinson’s disease, cerebrovascular disease, hemiplegia/paraplegia, or dementia, and a “0” if they did not have a diagnosis for one of these conditions). Clinicians hypothesized that higher age would be associated with greater impairment in everyday activities considered in TETRAS ADL. Diagnoses of anxiety and depression were included to account for potential differences in patients’ self-assessment of tremor-related impairment, as well as the ADL-scale component directly related to tremor-related anxiety and embarrassment. Finally, clinicians indicated that comorbidities affecting movement and balance could lead to additional impairment in ADL.

Association between upper limb tremor or ADL and QoL

For core regression models, five patient-level covariates identified based on clinician inputs included age, an indicator for current diagnosis of depression, an indicator for current diagnosis of anxiety, CCI, and BMI. Clinicians expected that all included covariates in the core model had the potential to correlate with worse QoL.

Sensitivity model specifications

The sensitivity analyses incorporated additional variables including sex, treatment status (currently treated, previously treated, never treated), alcohol use to alleviate tremor symptoms (across 6 frequency categories), body mass index (BMI), and Charlson Comorbidity Index (CCI). Indicators of treatment status and alcohol use to alleviate tremor symptoms were incorporated due to their potential impact on symptoms (or perception of symptoms), as well as the possibility that treatment could be prescribed more often to patients with greater ADL impairment (across varying levels of ET tremor amplitude). Inclusion of BMI was intended to account for potential movement difficulties stemming from obesity, while sex was added as an additional demographic control. CCI was included to control for impact on TETRAS ADL impairment scores related to a patient’s overall comorbidity profile. For regressions with EQ-5D index score as the dependent variable, a two-part generalized linear modelling (GLM) framework was also considered (recommended by ISPOR for EQ-5D-related regression analysis). Two-part models first estimate the probability (using a probit model) that the EQ-5D decrement (1 minus the EQ-5D score) is 0 (equivalent to an EQ-5D score of 1 prior to transformation) and subsequently fit a GLM (assuming a gamma distribution of errors and a log link function) to estimate the magnitude of EQ-5D decrements in the case where the score is not at the boundary.

For regressions with QUEST total score as the dependent variable, models assuming a gamma distribution of responses were also estimated.

Supplementary results

Upper limb tremor and ADL

Expanded model results with additional regression covariates (sex, treatment status, alcohol use to alleviate tremor symptoms, BMI, and CCI) demonstrated an association between TETRAS Performance Item 4 score and TETRAS ADL Total score (**Supplementary Table 3**), similar to the core regression model. A 1-point higher TETRAS Performance Item 4 score was associated with a 1.38-point higher mean TETRAS ADL Total score (P<0.01). The covariates of female sex and patients currently treated for ET were significantly associated with increased ADL Total score (both, P<0.01) (**Supplementary Table 3**). Core multivariable model results with physician-level RE were similar to core results with physician-level FE in the random intercept models (**Supplementary Table 3**).

Upper limb tremor and generic QoL

Expanded model results with additional regression covariates (sex, household income, an indicator for moderate-to-severe treatment-related adverse effects) demonstrated an association between TETRAS Performance Item 4 score and EQ-5D-5L Index score (**Supplementary Table 5**), similar to the core regression model. A 1-point higher TETRAS Performance Item 4 score was associated with a 0.0143-point lower mean EQ-5D-5L Index score (P<0.01). The covariate of moderate-to-severe treatment-related adverse effects was significantly associated with EQ-5D Index score (P<0.05) (**Supplementary Table 5**). Estimates from the two-part model for core and expanded multivariable regression models were comparable to those estimated using the linear model (**Supplementary Table 5**).

ADL and generic QoL

Expanded model results with additional regression covariates (sex, household income, an indicator for moderate-to-severe treatment-related adverse effects) demonstrated an association between TETRAS ADL Total score and EQ-5D-5L Index score (**Supplementary Table 6**), similar to the core regression model. A 1-point higher TETRAS ADL Total score was associated with a 0.0098-point lower mean EQ-5D-5L Index score (P<0.01). Estimates from the two-part model for TETRAS ADL were comparable to those estimated using the linear model (**Supplementary Table 6**).

Upper limb tremor and ET-specific QoL

Expanded model results with additional regression covariates (sex, household income, an indicator for moderate-to-severe treatment-related adverse effects) demonstrated an association between TETRAS Performance Item 4 score and QUEST Total score (**Supplementary Table 7**), similar to the core regression model. A 1-point higher TETRAS Performance Item 4 score was associated with a 1.652-point higher mean QUEST Total score (P<0.01). The covariates of patient-reported moderate-to-severe treatment-related adverse events (P<0.01) and household

income in the \$50k to \$100k range (*P*<0.05) were significantly associated with increased QUEST Total score (**Supplementary Table 7**).

ADL and ET-specific QoL

Expanded model results with additional regression covariates (sex, household income, an indicator for moderate-to-severe treatment-related adverse effects) demonstrated an association between TETRAS ADL Total score and QUEST Total score (**Supplementary Table 8**), similar to the core regression model. A 1-point higher TETRAS ADL Total score was associated with a 1.149-point higher mean QUEST Total score (*P*<0.01).

Supplementary Tables

Supplementary Table 1. Demographic and clinical characteristics of the study population

**Characteristic**	**All patients (*N*=1,003)**[1]	**All patients with nonmissing EQ-5D-5L Index scores (*N*=463)**[2]	**Patients with nonmissing QUEST Total scores (*N*=456)**[3]
BMI, mean (SD)	26.6 (4.6)	26.9 (4.6)	26.9 (4.6)
Insurance coverage, % (*n*)			
Medicare	50.0 (501)	48.8 (226)	47.6 (217)
Medicaid	5.3 (53)	3.9 (18)	3.7 (17)
Commercial	40.2 (403)	44.3 (205)	45.6 (208)
Other	4.6 (46)	3.0 (14)	3.1 (14)
Employment status, % (*n*)[4]			
Full-time	31.1 (312)	33.0 (153)	34.0 (155)
Part-time	9.6 (96)	9.5 (44)	9.4 (43)
Retired	46.2 (463)	43.2 (200)	42.3 (193)
Unemployed	12.5 (125)	13.8 (64)	13.8 (63)
Student	0.7 (7)	0.4 (2)	0.4 (2)
Current home circumstances, % (*n*)			
Residing at a nursing home	1.6 (16)	1.7 (8)	1.8 (8)
Residing with family	81.9 (821)	83.6 (387)	83.3 (380)
Residing alone	14.7 (147)	12.7 (59)	12.9 (59)
Other	0.6 (6)	0.9 (4)	0.9 (4)
Unknown	1.3 (13)	1.1 (5)	1.1 (5)
Has someone responsible for daily needs, % (*n*)			
Yes	23.9 (240)	35.0 (162)	34.2 (156)
No	71.8 (720)	60.9 (282)	61.6 (281)
Unknown	4.3 (43)	4.1 (19)	4.2 (19)
Annual household income			
$50k or less	—	23.1 (107)	23.2 (106)
$50k–$75k	—	15.6 (72)	15.4 (70)
$75k–$100k	—	16.2 (75)	16.9 (77)
$100k—$125k	—	6.9 (32)	7.2 (33)
$125k–$150k	—	7.6 (35)	7.7 (35)
$150k+	—	7.8 (36)	7.2 (33)
Did not report	—	22.9 (106)	22.4 (102)
**ET treatment/procedures**			
ET treatment status/history, % (*n*)			
Alcohol used to alleviate ET symptoms, % (*n*)			
Never	38.6 (387)	44.1 (204)	44.5 (203)
Monthly or less	21.3 (214)	17.7 (82)	17.8 (81)

2–4 times per month	16.0 (160)	12.3 (57)	12.1 (55)
2–3 times per week	9.6 (96)	10.6 (49)	10.7 (49)
4+ times per week	4.1 (41)	5.0 (23)	4.8 (22)
Don't know	10.5 (105)	10.4 (48)	10.1 (46)
Cannabis used to alleviate ET symptoms, % (*n*)			
Never	74.1 (743)	74.3 (344)	74.3 (339)
Monthly or less	5.6 (56)	6.9 (32)	6.8 (31)
2–4 times per month	2.9 (29)	1.9 (9)	2.0 (9)
2–3 times per week	1.4 (14)	1.5 (7)	1.5 (7)
4+ times per week	1.3 (13)	1.1 (5)	0.9 (4)
Don't know	14.8 (148)	14.3 (66)	14.5 (66)

1. Table reports patient characteristics for all patients with data derived from standardized clinician reports in the Adelphi ET DSP™ (*N*=1,003).
2. Table reports patient characteristics for all patients with data derived from patient-reported surveys in the Adelphi ET DSP™ with a nonmissing EQ-5D-5L Index score (*N*=463).
3. Table reports patient characteristics for all patients with data derived from patient-reported surveys in the Adelphi ET DSP™ with a nonmissing QUEST Total score (*N*=456).
4. In instances where responses differed between the standardized clinician reports and patient-reported surveys, responses from patient-reported questionnaires were utilized.

BMI, body mass index; DSP, Disease Specific Program; EQ-5D-5L, EuroQol-5 Dimension 5 Level; ET, essential tremor; QUEST, Quality of Life in Essential Tremor Questionnaire; SD, standard deviation.

Supplementary Table 2. Bivariate analyses for the associations of TETRAS ADL total score with TETRAS Performance Item 4 score and patient-level covariates

Variable	*N*	Correlation Coefficient	Mean	*P* value
TETRAS Performance Item 4 score[1]	1,003	0.761	–	<0.001
Age[1]	1,003	0.242	–	<0.001
BMI[1]	1,003	0.052	–	0.099
Female[2]	476	–	16.599	0.336
Male[2]	527	–	16.019	
Concomitant anxiety [2]	244	–	19.779	<0.001
No concomitant anxiety[2]	759	–	15.174	
Concomitant depression[2]	147	–	21.374	<0.001
No concomitant depression[2]	856	–	15.422	
Concomitant balance/movement comorbidity[2]	74	–	22.851	<0.001
No concomitant balance/movement comorbidity[2]	929	–	15.772	
Previously received treatment for ET[3]	73	–	17.493	<0.001
Currently receiving treatment for ET[3]	838	–	16.955	

Never received treatment for ET[3]	92	–	9.326	
Alcohol use to treat ET				
4+ times per week[3]	41	–	20.049	0.013
2–3 times per week[3]	96	–	17.625	
2–4 times per month[3]	160	–	16.463	
Monthly or less[3]	214	–	15.682	
Unknown[3]	105	–	14.219	
Never[3]	387	–	16.398	
CCI[1]	1,003	0.206	–	<0.001

Note: Key dependent and independent variables for bivariate analyses were based on review of survey questions and guidance from two clinicians with specialization in movement disorders.

1. For variables with continuous data, Pearson correlation tests were conducted to measure the strength of the linear relationship with primary outcome variables.
2. For variables with binary categorical data, Welch two-sample t-tests were conducted to test the null hypothesis that primary outcome means were equal across categories.
3. For variables with multicategorical data, one-way ANOVA tests were conducted to test the null hypothesis that primary outcome means were equal across categories.

ADL, activities of daily living; ANOVA, analysis of variance; BMI, body mass index; CCI, Charlson comorbidity index; ET, essential tremor; TETRAS, The Essential Tremor Rating Assessment Scale.

Supplementary Table 3. Expanded multivariable analyses for the associations of TETRAS ADL total score with TETRAS Performance Item 4 score and patient-level covariates

**Variable**	Core FE (robust SE)	Core RE (robust SE)	Expanded FE (robust SE)	Expanded RE (robust SE)
TETRAS Performance Item 4 score	1.42*** (0.070)	1.42*** (0.062)	1.38*** (0.071)	1.39*** (0.063)
Age	0.09*** (0.019)	0.08*** (0.018)	0.08*** (0.018)	0.07*** (0.017)
BMI	–	–	-0.02 (0.044)	-0.02 (0.041)
Female	–	–	0.98*** (0.349)	1.06*** (0.352)
Anxiety	-0.13 (0.422)	0.12 (0.427)	-0.12 (0.426)	0.15 (0.423)
Depression	2.16*** (0.695)	2.29*** (0.639)	1.97*** (0.679)	2.12*** (0.629)
Balance/movement comorbidity	2.53*** (0.642)	2.59*** (0.646)	2.55*** (0.619)	2.58*** (0.616)
Previously treated for ET (vs. never)	–	–	1.64 (0.986)	1.97** (0.962)
Currently treated for ET (vs. never)	–	–	2.14*** (0.581)	2.23*** (0.562)
Alcohol use to treat ET 2–3 times per week (vs. never)	–	–	-0.11 (0.888)	0.24 (0.847)
Alcohol use to treat ET 2–4 times per month (vs. never)	–	–	0.05 (0.654)	0.19 (0.608)
Alcohol use to treat ET 4+ times per week (vs. never)	–	–	1.24 (1.264)	1.48 (1.204)
Alcohol use to treat ET monthly or less (vs. never)	–	–	-0.25 (0.433)	-0.29 (0.455)
Unknown alcohol use to treat ET (vs. never)	–	–	-1.45**	-1.27**

			(0.712)	(0.636)
CCI	–	–	0.26<br>(0.160)	0.30<br>(0.155)
Constant	-3.14**<br>(1.200)	-3.07***<br>(1.113)	-3.83**<br>(1.544)	-4.18***<br>(1.500)
Observations	1,003	1,003	1,003	1,003
Number of physicians	98	98	98	98
Overall R-squared value	0.613	0.614	0.625	0.629
F-value	120.2	–	54.39	–
Wald's Chi-squared value	–	705.6	–	1011.0

****P*<0.01, ***P*<0.05.

ADL, activities of daily living; BMI, body mass index; CCI, Charlson comorbidity index; ET, essential tremor; FE, fixed effects; RE, random effects; SE, standard error; TETRAS, The Essential Tremor Rating Assessment Scale.

Supplementary Table 4. Bivariate analyses for associations of EQ-5D-5L index score and QUEST total score with TETRAS Performance Item 4 score, ADL total score, and patient-level covariates

Variable	*N*	Correlation coefficient	Mean	*P* value
**EQ-5D-5L index score**				
TETRAS Performance total score[1]	463	-0.475	–	<0.001
TETRAS Performance Item 4 score[1]	463	-0.410	–	<0.001
TETRAS ADL total score	463	-0.543	–	<0.001
Age[1]	463	-0.289	–	<0.001
BMI[1]	463	-0.093	–	0.046
Female[2]	219	–	0.728	0.223
Male[2]	244	–	0.752	
Race[3]				
White	370	–	0.749	0.386
African American	45	–	0.731	
Asian	13	–	0.716	
Hispanic	18	–	0.715	
Other	17	–	0.650	
Concomitant anxiety[2]	145	–	0.661	<0.001
No concomitant anxiety[2]	318	–	0.778	
Concomitant depression[2]	79	–	0.614	<0.001
No concomitant depression[2]	384	–	0.767	
CCI[1]	463	-0.197	–	<0.001
Moderate-to-severe treatment side effects[2]	85	–	0.653	<0.001
Mild or no treatment side effects[2]	378	–	0.761	
Household Income[3]				
$50k or less	107	–	0.709	0.242
$50k–$75k	72	–	0.742	
$75–$100k	75	–	0.760	
$100k–$125k	32	–	0.769	

$125k–$150k	35	–	0.771	
$150k+	36	–	0.799	
Did not report	106	–	0.720	
**QUEST total score**				
TETRAS Performance total score[1]	456	0.566		<0.001
TETRAS Performance Item 4 score[1]	456	0.457		<0.001
TETRAS ADL total score	456	0.630		<0.001
Age[1]	456	0.126		0.007
BMI[1]	456	0.048		0.306
Female[2]	219	–	26.366	0.053
Male[2]	237	–	22.880	
Race[3]				
White	364	–	23.224	0.036
African American	45	–	27.280	
Asian	12	–	32.917	
Hispanic	18	–	32.534	
Other	17	–	31.471	
Concomitant anxiety [2]	144	–	32.179	<0.001
No concomitant anxiety[2]	312	–	21.035	
Concomitant depression[2]	80	–	37.781	<0.001
No concomitant depression[2]	376	–	21.740	
CCI[1]	456	0.140		0.003
Moderate-to-severe treatment side effects[2]	85	–	35.299	<0.001
Mild or no treatment side effects[2]	371	–	22.092	
Household income[3]				
$50k or less	106	–	27.881	0.005
$50k–$75k	70	–	27.478	
$75–$100k	77	–	25.808	
$100k–$125k	33	–	28.427	
$125k–$150k	35	–	20.464	

$150k+	33	–	15.612	
Did not report	102	–	21.187	

Note: Key dependent and independent variables for bivariate analyses were based on review of survey questions and guidance from two clinicians with specialization in movement disorders.
1. For variables with continuous data, Pearson correlation tests were conducted to measure the strength of the linear relationship with primary outcome variables.
2. For variables with binary categorical data, Welch two-sample t-tests were conducted to test the null hypothesis that primary outcome means were equal across categories.
3. For variables with multicategory data, one-way ANOVA tests were conducted to test the null hypothesis that primary outcome means were equal across categories.
ADL, activities of daily living; ANOVA, analysis of variance; BMI, body mass index; CCI, Charlson comorbidity index; EQ-5D-5L, EuroQol-5 Dimension 5 Level; QUEST, Quality of Life in Essential Tremor questionnaire; TETRAS, The Essential Tremor Rating Assessment Scale.

Supplementary Table 5. Expanded multivariable analyses for the associations of EQ-5D-5L index score with TETRAS Performance Item 4 score and patient-level covariates

**Variable**	Core OLS (robust SE)	Core two-part (robust SE)	Expanded OLS (robust SE)	Expanded two-part (robust SE)
TETRAS Performance Item 4 score	-0.0148*** (0.002)	-0.0152*** (0.003)	-0.0143*** (0.002)	-0.0147*** (0.003)
Age	-0.0032*** (0.001)	-0.0034*** (0.001)	-0.0030*** (0.001)	-0.0033*** (0.001)
Female	–	–	-0.0132 (0.018)	-0.0189 (0.018)
BMI	-0.0019 (0.002)	-0.0011 (0.002)	-0.0027 (0.002)	-0.0020 (0.002)
Anxiety	-0.0456** (0.021)	-0.0524** (0.023)	-0.0389 (0.020)	-0.0417** (0.021)
Depression	-0.0942*** (0.032)	-0.0829*** (0.028)	-0.0933*** (0.031)	-0.0794*** (0.027)
CCI	-0.0093 (0.008)	-0.0040 (0.007)	-0.0092 (0.008)	-0.0038 (0.006)
Moderate-to-severe treatment side effects (vs. mild or no treatment side effects)	–	–	-0.0518** (0.025)	-0.0538** (0.023)
Household income: <$50k (vs. not reported)	–	–	0.0353 (0.023)	0.0344 (0.023)
Household income: ≥$50k and <$100k (vs. not reported)	–	–	0.0241 (0.029)	0.0277 (0.028)
Household income: ≥$100k (vs. not reported)	–	–	0.0285 (0.025)	0.0346 (0.029)
Constant	1.1787*** (0.081)	–	1.1772*** (0.088)	–
Observations	463	463	463	463
R-squared value	0.275	–	0.287	–
F-value	19.18	–	16.06	–
Log-pseudolikelihood	–	-53.27	–	-44.13

****P*<0.01, ***P*<0.05.
BMI, body mass index; CCI, Charlson comorbidity index; EQ-5D-5L, EuroQol-5 Dimension-5 Level; OLS, ordinary least squares; SE, standard error; TETRAS, The Essential Tremor Rating Assessment Scale.

Supplementary Table 6. Expanded multivariable analyses for the associations of EQ-5D-5L index score with TETRAS ADL total score and patient-level covariates

Variable	Core OLS (robust SE)	Core two-part (robust SE)	Expanded OLS (robust SE)	Expanded two-part (robust SE)
TETRAS ADL total score	-0.0100***<br>(0.001)	-0.0096***<br>(0.001)	-0.0098***<br>(0.001)	-0.0093***<br>(0.001)
Age	-0.0025***<br>(0.001)	-0.0029***<br>(0.001)	-0.0025***<br>(0.001)	-0.0027***<br>(0.001)
Female	–	–	-0.0044<br>(0.017)	-0.0120<br>(0.016)
BMI	-0.0026<br>(0.002)	-0.0019<br>(0.002)	-0.0029<br>(0.002)	-0.0023<br>(0.002)
Anxiety	-0.0370<br>(0.020)	-0.0450**<br>(0.022)	-0.0322<br>(0.020)	-0.0363<br>(0.021)
Depression	-0.0693***<br>(0.026)	-0.0549**<br>(0.025)	-0.0718***<br>(0.026)	-0.0533**<br>(0.024)
CCI	-0.0031<br>(0.007)	0.0000<br>(0.006)	-0.0023<br>(0.008)	0.0011<br>(0.006)
Moderate-to-severe treatment side effects (vs. mild or no treatment side effects)	–	–	-0.0247<br>(0.027)	-0.0316<br>(0.025)
Household income: <$50k (vs. not reported)	–	–	0.0284<br>(0.022)	0.0274<br>(0.023)
Household income: ≥$50k and <$100k (vs. not reported)	–	–	0.0357<br>(0.026)	0.0455*<br>(0.025)
Household income: ≥$100k (vs. not reported)	–	–	0.0386<br>(0.025)	0.0404<br>(0.027)
Constant	1.1743***<br>(0.080)	–	1.1531***<br>(0.086)	–
Observations	463	463	463	463
R-squared value	0.353	–	0.359	–
F-value	28.66	–	18.61	–

Log-pseudolikelihood	–	-51.41	–	-41.78

***P<0.01, **P<0.05.

ADL, activities of daily living; BMI, body mass index; CCI, Charlson comorbidity index; EQ-5D-5L, EuroQol-5 Dimension 5 Level; OLS, ordinary least squares; SE, standard error; TETRAS, The Essential Tremor Rating Assessment Scale.

Supplementary Table 7. Expanded multivariable analyses for the associations of QUEST total score with TETRAS Performance Item 4 score and patient-level covariates

**Variable**	Core OLS (robust SE)	Core Gamma (robust SE)	Expanded OLS (robust SE)	Expanded Gamma (robust SE)
TETRAS Performance Item 4 score	1.720*** (0.23)	1.782*** (0.32)	1.652*** (0.22)	1.747*** (0.31)
Age	-0.002 (0.08)	-0.013 (0.11)	-0.012 (0.07)	-0.018 (0.10)
Female	–	–	1.619 (1.21)	1.954 (1.24)
BMI	-0.213 (0.19)	-0.319 (0.26)	-0.131 (0.19)	-0.224 (0.27)
Anxiety	3.305 (2.33)	3.868 (2.64)	3.016 (2.19)	3.837 (2.33)
Depression	11.581*** (2.93)	10.494*** (2.92)	10.374*** (2.94)	9.891*** (2.89)
CCI	0.863 (0.61)	1.186 (0.68)	1.056 (0.63)	1.365** (0.67)
Moderate-to-severe treatment side effects (vs. mild or no treatment side effects)	–	–	7.524*** (2.49)	7.619*** (2.76)
Household income: <$50k (vs. not reported)	–	–	1.200 (2.51)	0.114 (2.96)
Household income: ≥$50k and <$100k (vs. not reported)	–	–	5.246** (2.56)	4.088 (3.13)
Household income: ≥$100k (vs. not reported)	–	–	2.759 (2.50)	1.575 (3.19)
Constant	9.833 (8.25)	–	4.330 (8.52)	–
Observations	456	456	456	456
R-squared value	0.291	–	0.328	–
F-value	22.98	–	27.64	–

Deviance	–	244.99	–	236.75

***P<0.01, **P<0.05.

BMI, body mass index; CCI, Charlson comorbidity index; OLS, ordinary least squares; QUEST, Quality of Life in Essential Tremor questionnaire; SE, standard error; TETRAS, The Essential Tremor Rating Assessment Scale.

Supplementary Table 8. Expanded multivariable analyses for the associations of QUEST total score with TETRAS ADL total score and patient-level covariates

**Variable**	Core OLS (robust SE)	Core Gamma (robust SE)	Expanded OLS (robust SE)	Expanded Gamma (robust SE)
TETRAS ADL total score	1.201*** (0.09)	1.308*** (0.15)	1.149*** (0.09)	1.274*** (0.16)
Age	-0.081 (0.07)	-0.123 (0.12)	-0.080 (0.07)	-0.115 (0.11)
Female	–	–	0.677 (1.10)	1.508 (1.25)
BMI	-0.152 (0.19)	-0.204 (0.26)	-0.117 (0.19)	-0.152 (0.27)
Anxiety	2.231 (1.97)	2.870 (2.35)	2.211 (1.87)	2.924 (2.21)
Depression	8.566*** (2.44)	7.170** (2.97)	7.821*** (2.51)	6.704** (3.16)
CCI	0.130 (0.53)	0.342 (0.63)	0.259 (0.57)	0.556 (0.65)
Moderate-to-severe treatment side effects (vs. Mild or no treatment side effects)	–	–	4.294 (2.31)	5.401** (2.74)
Household income: <$50k (vs. not reported)	–	–	1.988 (2.40)	0.912 (2.81)
Household income: ≥$50k and <$100k (vs. not reported)	–	–	3.989 (2.13)	2.710 (2.87)
Household Income: ≥$100k (vs. not reported)	–	–	1.707 (2.30)	2.047 (3.19)
Constant	10.664 (7.96)	–	7.325 (8.37)	–
Observations	456	456	456	456
R-squared value	0.438	–	0.452	–
F-value	54.16	–	52.35	–
Deviance	–	210.57	–	207.09

****P*<0.01, ***P*<0.05.
ADL, activities of daily living; BMI, body mass index; CCI, Charlson comorbidity index; OLS, ordinary least squares; QUEST, Quality of Life in Essential Tremor questionnaire; SE, standard error; TETRAS, The Essential Tremor Rating Assessment Scale.

Supplementary Figure 1. Visual representation of the Adelphi ET dataset

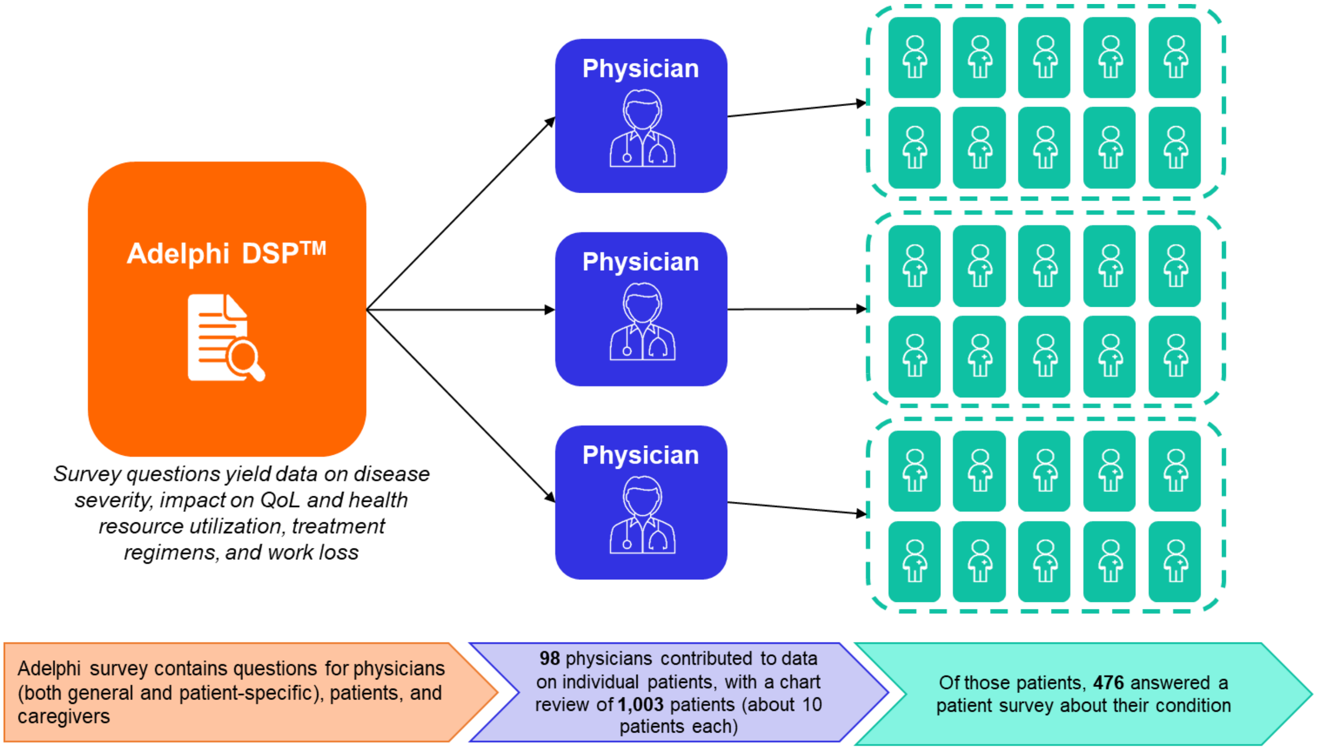

<u>Note:</u> Findings from the Adelphi survey data may not fully represent the universe of ET patients, physicians, and caregivers.
DSP, Disease Specific Programme; ET, essential tremor; QoL, quality of life.
